# Differential Gene Expression in Circulating CD14^+^ Monocytes Indicates the Prognosis of Critically Ill Patients with Sepsis

**DOI:** 10.3390/jcm9010127

**Published:** 2020-01-02

**Authors:** Anke Liepelt, Philipp Hohlstein, Hendrik Gussen, Jia Xue, Anna C. Aschenbrenner, Thomas Ulas, Lukas Buendgens, Klaudia T. Warzecha, Matthias Bartneck, Tom Luedde, Christian Trautwein, Joachim L. Schultze, Alexander Koch, Frank Tacke

**Affiliations:** 1 Department of Medicine III, University Hospital Aachen, RWTH Aachen University, 52074 Aachen, Germany; liepelt.anke@gmail.com (A.L.); phohlstein@ukaachen.de (P.H.); hgussen@ukaachen.de (H.G.); lbuendgens@ukaachen.de (L.B.); klaudia.kaczmarski@rwth-aachen.de (K.T.W.); mbartneck@ukaachen.de (M.B.); tluedde@ukaachen.de (T.L.); ctrautwein@ukaachen.de (C.T.); akoch@ukaachen.de (A.K.); 2 Department of Anaesthesiology, University Hospital Aachen, RWTH Aachen University, 52074 Aachen, Germany; 3 Genomics and Immunoregulation, Life & Medical Sciences (LIMES) Institute, University of Bonn, 53115 Bonn, Germany; jiaxue@uni-bonn.de (J.X.); a.aschenbrenner@uni-bonn.de (A.C.A.); t.ulas@uni-bonn.de (T.U.); j.schultze@uni-bonn.de (J.L.S.); 4 Department of Internal Medicine and Radboud Center for Infectious Diseases (RCI), Radboud University Medical Center, 6525 Nijmegen, The Netherlands; 5 Department of Hepatology & Gastroenterology, Campus Virchow Klinikum and Charité Campus Mitte, Charité University Medicine Berlin, 13353 Berlin, Germany

**Keywords:** ICU, sepsis, mortality, prognosis, innate immunity, monocytes, biomarker

## Abstract

Critical illness and sepsis are characterized by drastic changes in the systemic innate immune response, particularly involving monocytes. The exact monocyte activation profile during sepsis, however, has remained obscure. Therefore, we prospectively analyzed the gene expression profile of circulating CD14^+^ monocytes from healthy volunteers (*n* = 54) and intensive care unit (ICU) patients (*n* = 76), of which *n* = 36 had sepsis. RNA sequencing of selected samples revealed that monocytes from septic ICU patients display a peculiar activation pattern, which resembles characteristic functional stages of monocyte-derived macrophages and is distinct from controls or non-sepsis ICU patients. Focusing on 55 highly variable genes selected for further investigation, arachidonate 5-lipoxygenase-activating protein (ALOX5AP) was highly upregulated in monocytes of ICU patients and only normalized during 7 days in the ICU in non-sepsis patients. Strikingly, low monocytic guanine nucleotide exchange factor 10-like protein (ARHGEF10L) mRNA expression was associated with the disease severity and mortality of ICU patients. Collectively, our comprehensive analysis of circulating monocytes in critically ill patients revealed a distinct activation pattern, particularly in ICU patients with sepsis. The association with disease severity, the longitudinal recovery or lack thereof during the ICU stay, and the association with prognosis indicate the clinical relevance of monocytic gene expression profiles during sepsis.

## 1. Introduction

Sepsis is a leading cause of death worldwide. It often manifests as a complex immune dysfunction where a hyper-inflammatory reaction, the “cytokine storm”, simultaneously occurs alongside an insufficient (“hypo”) immune reaction [1,2]. Several studies have demonstrated that persistent immunosuppression is associated with a poor patient outcome [3,4]. The anti-inflammatory response may explain persistent and/or nosocomial infections, organ failure, and high mortality. It is estimated that there are 31.5 million cases of sepsis and 19.4 million cases of severe sepsis worldwide every year, potentially leading to 5.3 million deaths [5]. The early diagnosis of sepsis is critical in order to initiate effective therapeutic strategies. Therefore, reliable biomarkers are necessary to guide treatment decisions. Currently, microbiological cultures, serum C-reactive protein (CRP), procalcitonin (PCT), presepsin, and interleukin-6 (IL-6) are widely used as such markers [6,7]. Additionally, low serum 25-hydroxyvitamin D levels have been shown to impact the outcome of critically ill patients with sepsis [8,9]. Reliable biomarkers provide at least two major advances in the difficult clinical management of sepsis: early recognition and differential etiological diagnosis. In fact, circulating microRNAs are very sensitive and early markers, so their use might have a relevant impact [10,11]. In particular, miR-15a and miR-16 are upregulated in the serum of neonatal sepsis patients [12] and, furthermore, have been shown to be useful to distinguish patients with sepsis from those with systemic inflammatory response syndrome (SIRS) [13]. Other microRNAs, such as miR-133a, might even have predictive power in the setting of critical illness [14].

Patients with sepsis typically display multiple perturbations of the immune system, like abnormal leukocyte numbers and functional alterations of inflammatory responses. Among these multiple adaptations, the inflammatory status during sepsis has been closely linked to the functional status (“polarization”) and cellular metabolism of myeloid cells, particularly monocytes and macrophages [15]. In a prospective study comprising more than 100 critically ill patients admitted to the ICU, we recently demonstrated a significant increase in total leukocyte numbers in the peripheral blood of ICU patients compared to healthy volunteers and patients with infections from a standard care unit. At the same time, lymphocyte numbers were decreased, which was associated with increased mortality [16].

Concerning innate immunity, several reports demonstrate that the numbers of circulating monocytes are increased in patients with diagnosed sepsis [17,18,19]. In humans, monocytes are subdivided into at least three different populations according to their surface expression of the LPS-co-receptor CD14 and the Fcγ receptor CD16. The major subpopulation is comprised of the CD14^+^CD16^−^ monocytes, or classical monocytes, and the minor population is comprised of the CD14^−^CD16^+^ non-classical monocytes. An intermediate population is characterized by a high expression of both CD14 and CD16 (CD14^+^CD16^+^ monocytes) [20]. A recent study employing single cell RNA sequencing revealed the presence of another, probably intermediate, monocytic subpopulation [21]. Some reports indicate a contribution of the different monocyte subpopulations to the pathogenesis of sepsis. Work from Mukherjee and colleagues elucidated a decrease of classical monocytes in septic patients compared to healthy controls, while the non-classical and intermediate monocyte populations are increased [22]. The same study also suggested a more inflammatory phenotype of the non-classical and intermediate monocytes with a high expression of CD80, CD86, and human leukocyte antigen-DR isotype (HLA-DR) [22]. In terms of total circulating monocytes, the surface expression of HLA-DR, an major histocompatibility complex (MHC) class II molecule, is reduced in septic patients, and the reduced HLA-DR expression correlates with a poor outcome [19,23,24]. Another surface marker associated with the survival of septic patients is the fractalkine receptor CX_3_CR1. The expression of CX_3_CR1 is downregulated on septic monocytes compared to healthy controls, and non-survivors sustainably express even lower amounts of this receptor [25].

The aim of our study was to assess functional alterations in circulating monocytes in patients with sepsis compared to non-septic patients and healthy individuals. In a first unbiased approach, we therefore isolated CD14^+^ monocytes from ICU patients with and without sepsis, respectively, as well as healthy donors, and comprehensively analyzed their transcriptome by RNA sequencing. In a validation and prognostic approach, 55 highly regulated genes were chosen for an analysis of isolated circulating monocytes of large patient cohorts, also comprising a diseased control population of standard care patients with confirmed infections. Compared to whole blood transcriptional profiling [26], our approach of the pre-selection of CD14^+^ monocytes allows the comprehensive analysis of inflammatory reactions of monocytes as key players of the innate immune system. This study will help to understand the nature and contribution of circulating monocytes in critically ill patients.

## 2. Experimental Section

### 2.1. Patients and Controls

This study was approved by the local ethics committee (EK 150/06) of the University Hospital Aachen, RWTH Aachen University, and written informed consent was obtained from every participant or authorized relatives in the case of unconsciousness. Critically ill patients were prospectively included upon admission to the medical intensive care unit (ICU) and standard care (SC) wards of the Department of Medicine III of the University Hospital Aachen, following an established protocol [16,27,28] and using an enrollment process, as previously described [29,30]. Patients treated at the ICU because of sepsis had a clinically suspected or verified infection diagnosed by the intensive care physicians and were treated with antibiotics. Sepsis diagnosis was established following a diagnosed infection and an increase in the Sepsis-related Organ Failure Assessment (SOFA) score greater than or equal to two points [31]. Non-critically ill patients, admitted due to infectious diseases to the standard care ward, served as a diseased control population. Those patients were admitted to the hospital following a diagnosis of infection by the treating physician (based on clinical judgment, laboratory results, and/or microbial cultures) and received antibiotic therapy [16,30]. Samples from healthy volunteers were acquired from the local blood transfusion institute and served as a healthy control population. Blood samples of the recruited patients were obtained by peripheral venipuncture or from inlying central venous or arterial catheters at day 1 (admission), day 3, and day 7 of the ICU stay. To prevent the coagulation of blood samples, 250 units of heparin (Rotexmedica, Frittach, Germany) per milliliter blood were added to the samples [16,30]. Samples were processed directly after collection.

### 2.2. Isolation of Peripheral Blood Mononuclear Cells and Polymorphonuclear Cells

Blood and cells were kept at 4 °C during all procedures to care for minimal cell activation. Peripheral blood mononuclear cells (PBMC) were isolated using a Ficoll-based density gradient. Therefore, whole blood was mixed with an equal amount of phosphate-buffered saline (PBS, PAN Biotech, Aidenbach, Germany), and was subsequently carefully manually layered over 1077 Lymphocyte Separation Medium (PAA, Pasching, Austria), followed by centrifugation at 1600 rpm for 40 min without the use of a brake at room temperature. The intermediate layer containing the PBMC was then carefully harvested, washed with PBS, and centrifuged at 1300 rpm for 10 min three times. In the last step, the cells were resuspended in PBS and counted using a Neubauer chamber as a preparation step for antibody staining and Magnetic-activated cell sorting (MACS) [16,30]. Polymorphonuclear cells were isolated as described before [30] using 5% dextran in PBS at 37 °C for 45 min (500,000 dextran, Merck KGaA, Darmstadt, Germany). The upper phase containing leukocytes was transferred into a new tube, and osmotic lysis of red blood cells was done by incubation for 20 s in distilled water and recovery using 10× PBS.

### 2.3. Flow Cytometry

Two million cells were resuspended in PBS and blocking buffer (2% bovine serum albumin, 2% rabbit serum, 2% human serum, 2% mouse serum, and 2% rat serum) to reduce unspecific binding and stained with fluorescence-conjugated antibodies against CD14, CD56, CD45, CX_3_CR1, HLA-DR (eBioscience; San Diego, CA, USA), and CD16 (BD, Heidelberg, Germany). Cells were then subjected to flow-cytometric analysis using a FACS Canto-II (BD, Heidelberg, Germany) and analyzed using FlowJo software (TreeStar Inc., Ashland, TN, USA). After the exclusion of doublets, monocytes were identified by the exclusion of CD56-positive cells and CD14 positivity, as well as their characteristic distribution in forward and sideward scatter, to ensure a clean population of monocytes. Subpopulations were defined by their respective expression of CD14 and CD16. Absolute cell numbers were calculated based on automated differential white blood cell counts.

### 2.4. Isolation of CD14^+^ Monocytes

At least 10^7^ cells were resuspended in MACS buffer (PBS, 2 mM EDTA, 0.5% BSA), incubated with CD14-Microbeads (Miltenyi Biotec, Bergisch Gladbach, Germany), and CD14^+^ cells were isolated according to standard protocols provided by the manufacturer. After isolation, CD14^+^ cells were directly stored in PeqGOLD Trifast (Peqlab, Erlangen, Germany) at −80 °C until further analysis.

### 2.5. Library Preparation

For RNA sequencing, five samples were randomly chosen from healthy donors, ICU patients with sepsis, and ICU patients without sepsis, respectively. RNA was isolated from cells stored in PeqGOLD according to standard protocols provided by the manufacturer. Total RNA was converted into libraries of double-stranded cDNA molecules as a template for high throughput sequencing following the manufacturer’s recommendations, using the Illumina TruSeq RNA Sample Preparation Kit v2. mRNA was purified from 100 ng of total RNA using poly-T oligo-attached magnetic beads. Fragmentation was carried out using divalent cations under an elevated temperature in Illumina proprietary fragmentation buffer. First-strand cDNA was synthesized using random oligonucleotides and SuperScript II. Second-strand cDNA synthesis was subsequently performed using DNA Polymerase I and RNase H. Remaining overhangs were converted into blunt ends via exonuclease/polymerase activities and enzymes were removed. After the adenylation of 3′ ends of DNA fragments, Illumina PE adapter oligonucleotides were ligated to prepare for hybridization. DNA fragments with ligated adapter molecules were selectively enriched using Illumina PCR primer PE1.0 and PE2.0 in a 15 cycle PCR reaction. Size-selection and purification of cDNA fragments with preferentially 75 bp in length were performed using SPRIBeads (Beckman-Coulter, Brea, CA, USA). The size-distribution of cDNA libraries was measured using the Agilent high-sensitivity DNA assay on a Bioanalyzer 2100 system (Agilent, Santa Clara, CA, USA). cDNA libraries were quantified using KAPA Library Quantification Kits (Kapa Biosystems, Wilmington, MA, USA). After cluster generation on a cBot, a 75 bp single-end run was performed on a HiSeq1500.

### 2.6. Standard Bioinformatic Analysis

The total number of reads ranged between 4,000,000 and 26,000,000. After base calling and de-multiplexing using CASAVA version 1.8, the 75 bp paired-end reads were aligned to the murine reference genome hg19 from UCSC by TopHat2 version v2.0.11 using the default parameters. This annotation included 19.225 unique transcript entries with genomic coordinates. After mapping the reads to the genome, we imported the data into Partek Genomics Suite V6.6 (PGS) to calculate the number of reads mapped to each transcript against the RefSeq hg19 annotation download on May 2015. These raw read counts were used as the input to DESeq2 for the calculation of normalized signals for each transcript using the default parameters. After DESeq2 normalization, the normalized read counts were imported back into PGS and floored by setting all read counts to at least a read count of 1 after the batch-correction. Subsequent to flooring, all transcripts having a maximum overall group mean lower than 10 were removed. After dismissing the low expressed transcripts, the data comprised 10.209 transcripts. RNA-seq data can be accessed under GSE139913.

To visualize the structure within the data, we performed Principle Component Analysis (PCA) on all genes with default settings in PGS. Additionally, co-regulation analysis (CRA) based on Pearson’s correlation coefficients for the samples using BioLayout Express3D [32] was performed to describe the structure within the data set. For enrichment maps, differentially expressed genes were selected by an ANOVA *p*-value threshold of ≥0.05 and a fold change of at least 2 or −2, respectively. Selected differentially expressed genes were subjected to GOEA (gene ontology (GO) biological process) by using the Cytoscape [33] plug-in BinGO [34]. For network visualization, the Cytoscape plugin Enrichment Map [35] was used. For enrichment map visualization, the following criteria were chosen: *p*-value ≥ 0.05, False Discovery Rate (FDR) Q-value cutoff ≥ 0.2, and similarity cutoff with Jaccard coefficient ≥ 0.5. Principal Component Analysis (PCA) revealed two outliers (one sepsis and one non-sepsis ICU patient). During construction of the correlation network using the defined correlation cut-off criteria, those two samples were not connected to their respective group. Therefore, they were excluded for further analysis, following previously established algorithms.

### 2.7. Preparation of RNA and NanoString Analysis

RNA for NanoString analysis was isolated from cells stored in PeqGOLD according to standard protocols provided by the manufacturer, with 40 µg Glycogen (ThermoScientific, Dreieich, Germany) being added to improve RNA precipitation. A total of 100 ng RNA was used for analysis with the predefined panel on a nCounter System (NanoString, Seattle, WA, USA). Five mRNAs with the lowest variation among the three groups from RNA sequencing data were selected as housekeeping genes.

The normalization and generation of transcript counts was conducted by employing NanoString nSolver 3.0 (NanoString, Seattle, WA, USA) and the R package DESeq2 [36] using the geometric mean values of the five housekeeping mRNAs. For principal component analysis, the respective function from DESeq2 was used. Heatmaps were generated using the R package gplots with the heatmap.2 function and the hierarchical clustering method “complete”.

### 2.8. Statistical Analysis

Data were analyzed using SPSS (version 25, SPSS Inc., Chicago, IL, USA) and GraphPad Prism 5 (GraphPad Software Inc., La Jolla, CA, USA). As a normal distribution of samples could not be assumed, the Kruskal–Wallis test followed by post hoc testing by Dunn’s multiple comparison test was used for more than two groups, the two-tailed Mann–Whitney U test was used for two groups of unpaired samples, and the two-tailed Wilcoxon signed rank test was used for paired samples. A significance level of α = 0.05 was used in all corresponding calculations. The Youden index was calculated to identify the optimal cut-off values for parameters to discriminate prognosis [29]. Receiver operating characteristic (ROC) curve analysis and the derived area under the curve (AUC) statistics were generated by plotting sensitivity against 1-specificity [37]. Correlations between variables were assessed with Spearman rank correlation tests. Associations with survival were assessed by Cox regression, and patient survival was depicted by Kaplan–Meier curves.

## 3. Results

### 3.1. Alterations in the Composition of Circulating Monocyte Populations and Their Surface Marker Expression in Critically Ill Patients

Several studies reported increased numbers of circulating monocytes in patients with diagnosed sepsis [17,18], including alterations of the monocyte subset distribution [22]. We herein investigated the abundance and phenotype of different monocyte subpopulations in a prospective patient study, including 54 healthy controls (HC), 42 standard care patients (SC) with confirmed infection, and 76 critically ill patients upon admission to the intensive care unit (ICU) (Table 1).

**Figure 1:**
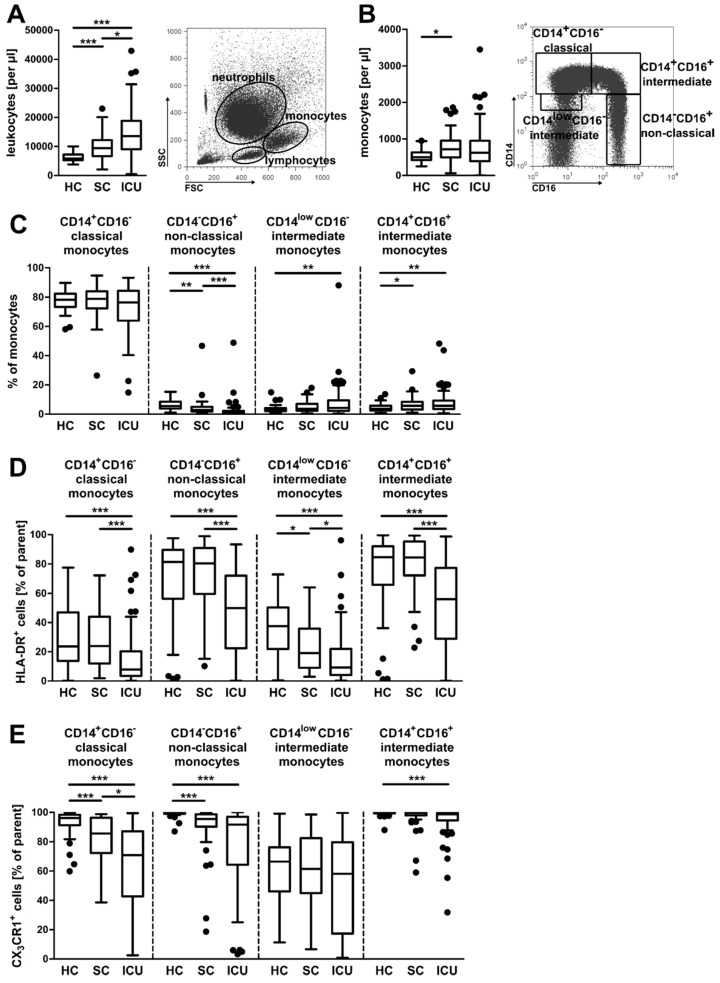
Monocyte subsets in healthy volunteers, standard care patients with infection, and intensive care patients. (**A**) Absolute numbers of circulating leukocytes (left panel) and a representative scatter plot of isolated leukocytes from whole blood (right panel). (**B**) Absolute numbers of circulating monocytes from the different patient cohorts (left panel), and a representative gating strategy for monocyte subpopulations (right panel, after the Ficoll density gradient). (**C**) Percentages of the four different monocyte subpopulations. (**D**,**E**) Percentages of human leukocyte antigen-DR isotype (HLA-DR^+^) cells (**D**) and fractalkine receptor (CX_3_CR1^+^) cells (**E**) of the four monocyte subpopulations. Statistics: * indicates *p* < 0.05, ** *p* < 0.01, and *** *p* < 0.001. For a comparison of more than two groups, the Kruskal–Wallis test was performed, followed by post hoc testing by Dunn’s multiple comparison test. Sample sizes: healthy controls (HC) *n* ≥ 52, standard care (SC) *n* = 42, and intensive care unit (ICU) *n* ≥ 74.

Peripheral blood leukocytes were analyzed by flow cytometry. As recently demonstrated by our group [16], the total number of circulating leukocytes was significantly increased in patients from the ICU and standard care compared to healthy controls (Figure 1A). The total number of monocytes was increased in patients from the ICU in comparison to healthy controls (Figure 1B, left panel). Peripheral monocytes were discriminated into four subpopulations based on their CD14 and CD16 surface expression (Figure 1B, right panel). Both relative and absolute numbers of CD14^+^CD16^−^ classical monocytes were not significantly altered between the patient cohorts (Figure 1C and Supplementary Materials Figure S1A). CD14^−^CD16^+^ non-classical monocytes were significantly decreased, while both intermediate monocyte subpopulations were significantly increased, when comparing HC and ICU groups (Figure 1C and Supplementary Materials Figure S1A). A reduced surface expression of HLA-DR is characteristic for monocytes in septic patients [23,24]. In our study, HLA-DR expression was indeed significantly decreased on each of the four monocyte subsets when comparing the ICU patients with the SC or healthy group, respectively (Figure 1D and Supplementary Materials Figure S1B). Expression of the fractalkine receptor (CX_3_CR1) on monocyte subtypes was also decreased (Figure 1E and Supplementary Materials Figure S1C), as has been described for monocytes in sepsis [25].

Among septic ICU patients, n = 8 (22.2% of 36) received statin therapy before the development of sepsis. There was no difference (*p* = 0.709) in survival rates among septic patients receiving (survival of 5 patients, 62.5%) or not receiving (survival of 15 patients, 53.6%) statin therapy.

**Figure 2:**
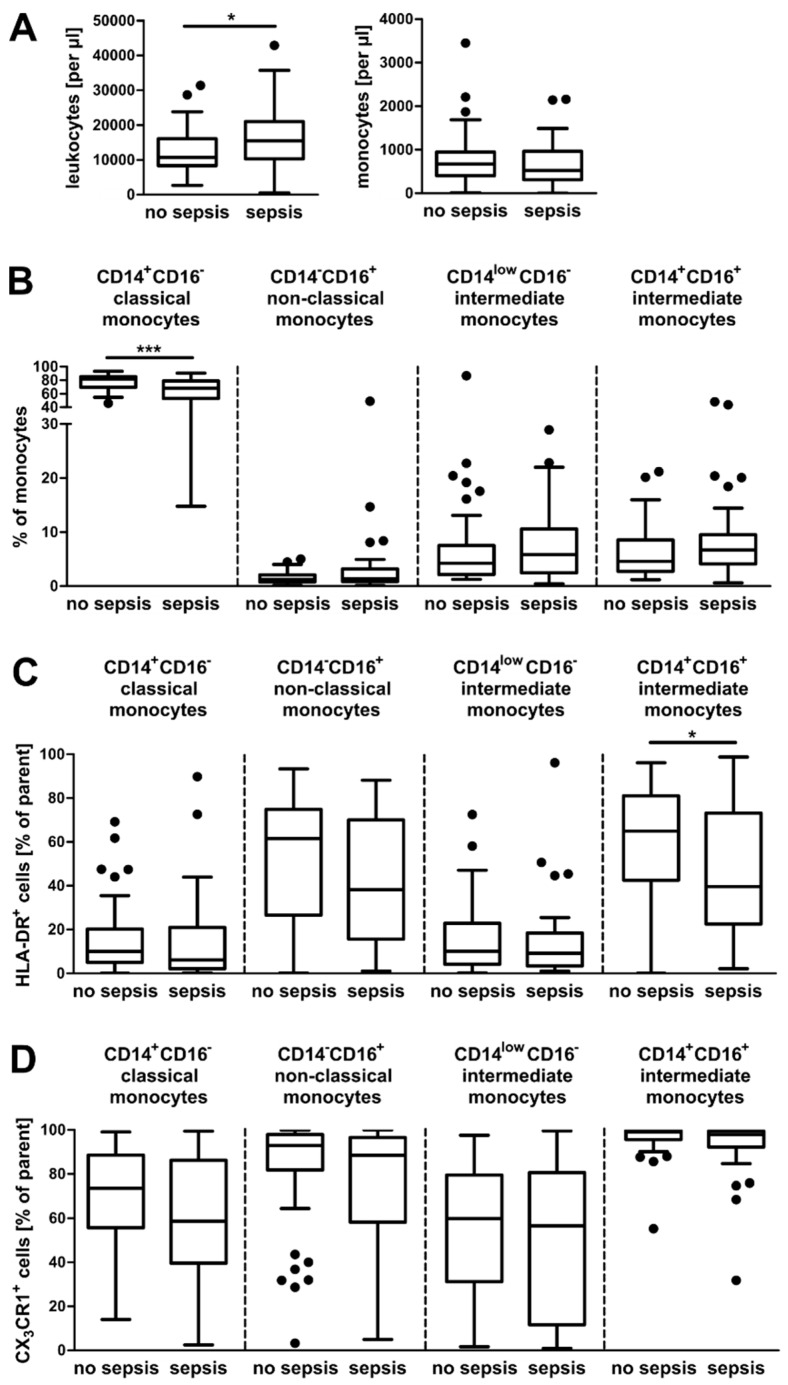
Monocyte subsets in ICU patients with and without sepsis. (**A**) Absolute numbers of circulating leukocytes and monocytes from the different patient cohorts. (**B**) Percentages of the four different monocyte subpopulations. (**C**,**D**) Percentages of HLA-DR^+^ cells (**C**) and CX_3_CR1^+^ cells (**D**) of the four monocyte subpopulations. Statistics: * indicates *p* < 0.05 and *** *p* < 0.001. For a comparison of two groups, the Mann–Whitney U test was used. Sample sizes: no sepsis *n* ≥ 38 and sepsis *n* = 36.

Contrary to other reports [17,18], the total numbers of circulating monocytes remain unchanged in our study between septic (*n* = 36) and non-septic (*n* = 40) ICU patients (Figure 2A, right panel). However, we could observe alterations in the composition of monocyte populations dependent on the presence of sepsis. Classical monocytes were significantly reduced in septic patients, whereas the other three populations tended to be increased in patients diagnosed with sepsis (Figure 2B and Supplementary Materials Figure S2A). These results are in line with a recently published study investigating smaller patient cohorts (*n* = 9 and *n* = 11) [22]. The expression of HLA-DR in monocytes from septic patients was reduced, confirming previous findings (Figure 2C and Supplementary Materials Figure S2B) [23,24]. We could also observe a slightly decreased expression of CX_3_CR1 on monocytes from patients with sepsis (Figure 2D and Supplementary Materials Figure S2C).

### 3.2. Gene Expression in CD14^+^ Monocytes from Critically Ill Patients

**Figure 3:**
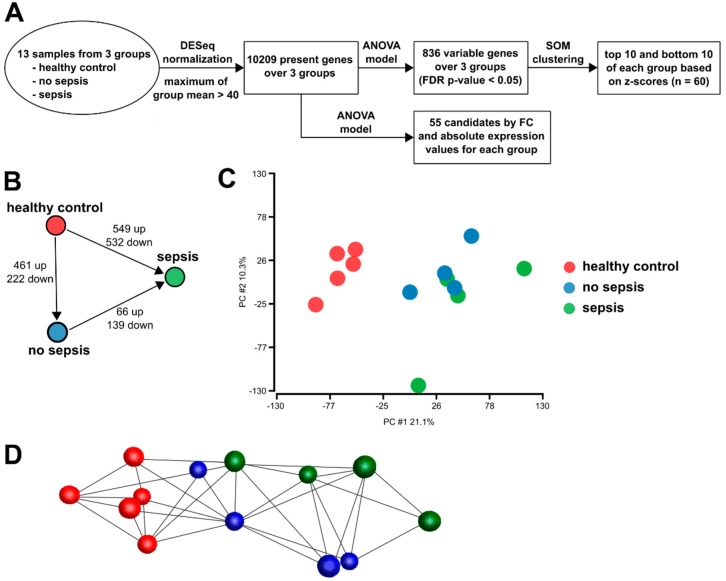
Data structure of monocyte RNA sequencing data. (**A**) Schematic approach of RNA sequencing data analysis and candidate mRNA selection for NanoString analysis. (**B**) Schematic representation of the number of differentially expressed genes between the three cohorts. (**C**) Principal component analysis (PCA) of the transcriptome data, depicting the group relationships of healthy controls, no sepsis, and sepsis. The proportion of component variance is indicated as a percentage. (**D**) Visualization of the sample to sample correlation of healthy control, no sepsis, and sepsis individuals.

To gain insight into the phenotypic differences of monocytes, we isolated CD14^+^ monocytes and analyzed their gene expression in an unbiased comprehensive approach by RNA sequencing. Samples from ICU patients with sepsis were compared with those from ICU patients without sepsis and age-matched healthy controls. Figure 3A depicts the strategy of bioinformatics analyses and Figure 3B shows the number of differentially expressed genes between the three patient cohorts. Principal component analysis revealed the close clustering of healthy control samples. Samples from ICU patients clustered together as well, irrespective of the sepsis (Figure 3C). The clustering of samples in a co-regulation network revealed two major clusters of patient groups (Figure 3D).

### 3.3. Enrichment of Activation Modules from Human Macrophages in Circulating Monocytes of ICU Patients

**Figure 4:**
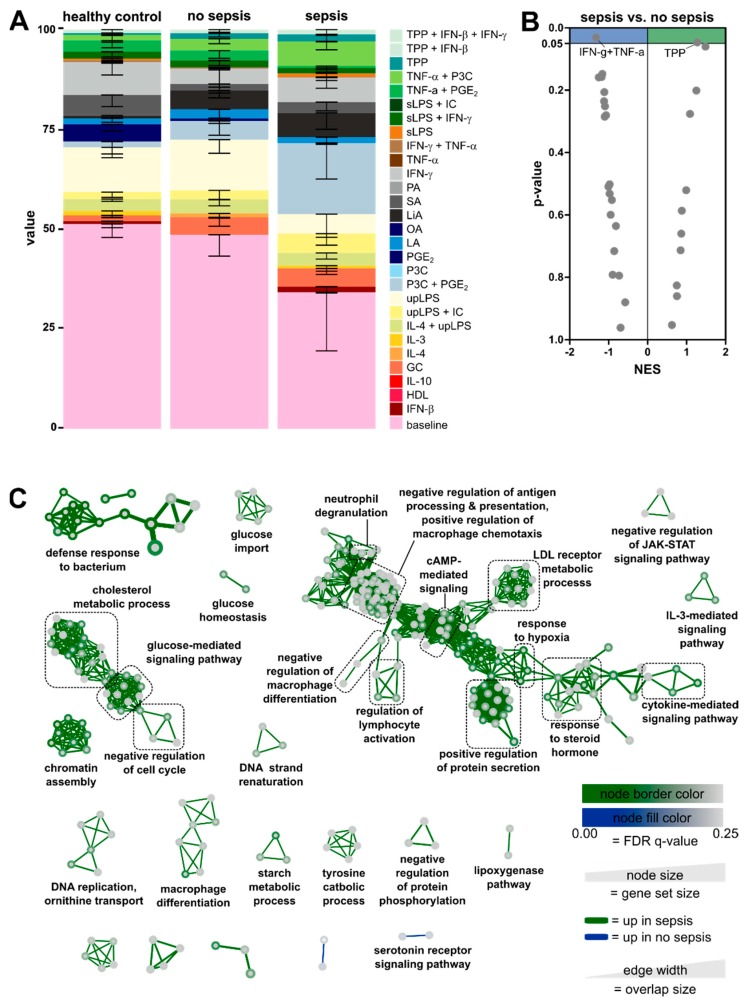
Enrichment of activation modules from monocyte-derived in vitro-differentiated human macrophages [38]. (**A**) Relative fractions of human monocyte-derived macrophage activation signatures from 28 activation conditions enriched in the three patient cohorts are visualized as a stacked bar plot. As a human macrophage activation signature matrix, 184 transcriptomes representing 29 conditions from the human macrophage activation resource data were used for CIBERSORT (IC, immune complexes; PA, palmitic acid; OA, oleic acid; LA, lauric acid; LiA, linoleic acid; SA, stearic acid; sLPS, standard lipopolysaccharide; HDL, high density lipoprotein). (**B**) Volcano plots of normalized enrichment scores (NES) and enrichment p-values comparing sepsis and non-sepsis based on Gene Set Enrichment Analysis (GSEA) using weighted gene co-expression network analysis (WGCNA) modules. (**C**) Enrichment Map Network visualization of differentially expressed genes from septic patients vs. patients without sepsis.

In order to relate the monocyte transcriptomic data from our cohorts to known human immune cell activation programs, we used the human macrophage activation signatures that had been generated from human in vitro-differentiated monocyte-derived macrophages activated by an array of defined stimuli, as previously described in [38]. Signatures from untreated baseline in vitro-differentiated macrophages, as well as 28 activation conditions, were linked to the data from the three patient groups using CIBERSORT (Figure 4A). In monocytes from healthy controls, characteristic genes from unstimulated baseline macrophages were the most prominently enriched signature. ICU patients without sepsis showed a monocyte transcriptome enriched in the baseline signature, as seen in healthy controls. Within the stimulation-specific signatures, the monocytes of ICU patients displayed a slight shift towards pro-inflammatory signatures (ultrapure lipopolysaccharide (upLPS), Pam_3_CysSerLys_4_/prostaglandin E2 (P3C + PGE_2_), TNF-α + P3C, and TNF-α/P3C/PGE_2_ (TPP)), while also showing slight enrichment of the glucocorticoid (GC) signature. Septic patients, however, displayed striking differences compared to non-septic patients and healthy controls. Most prominently, the baseline-related signature was less dominant and pro-inflammatory response signatures were enhanced. Enrichment shifted from an upLPS-associated stimulation profile to P3C + PGE_2_, IFN-γ, and TNF-α/P3C signatures. In order to better apprehend the differences between sepsis and non-sepsis patients, we performed Gene Set Enrichment Analysis (GSEA) (Figure 4B). In sepsis, a single module associated with TNF-α/P3C/PGE_2_ (TPP) was significantly enriched. In non-septic patients, a module associated with IFN-γ/TNF-α stimulation was enriched, suggesting a more classical activation (previously called M1) of these monocytes.

To functionally analyze the differences between non-sepsis and sepsis patients, we employed gene ontology (GO) pathway analysis of the differentially expressed genes (Figure 4C). Most significantly enriched GO pathways in septic patients were related to the defense response to the bacterium, reflecting the established infection in these patients. Furthermore, we observed an accumulation of pathways associated with metabolism, cell differentiation, and proliferation, as well as the immune response in patients with sepsis (Figure 4C). In patients without sepsis, fewer genes were regulated, and as a consequence, fewer pathways were enriched.

### 3.4. Targeted Gene Expression Analysis in Monocytes from Total Patient Cohorts

**Figure 5:**
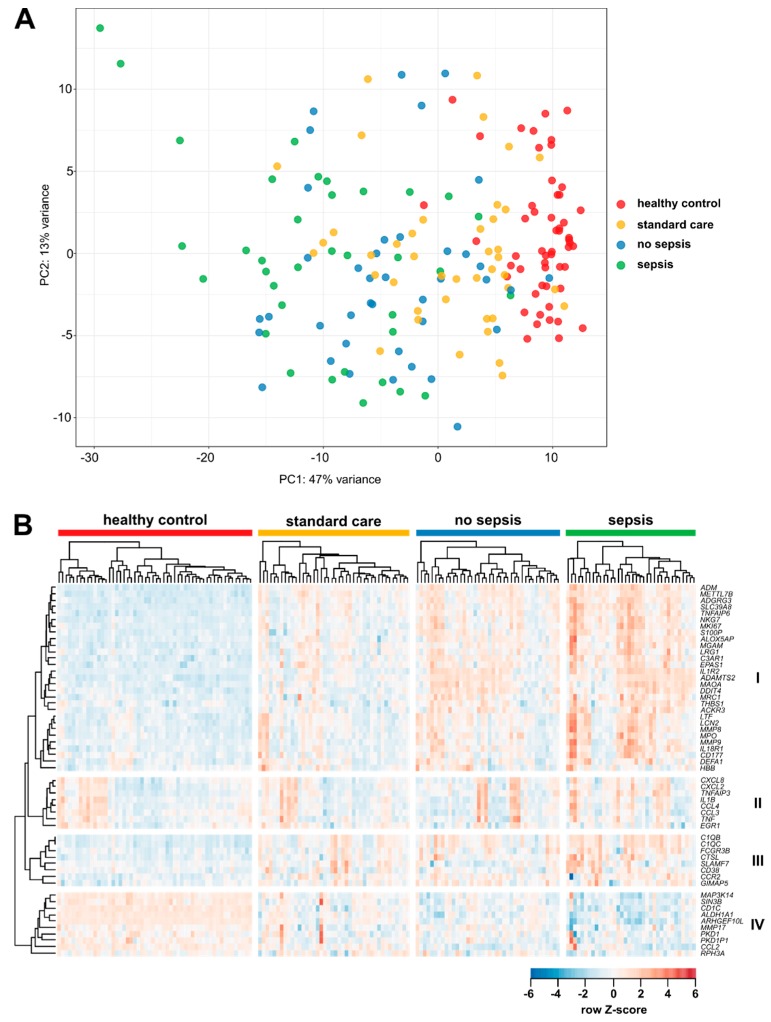
NanoString analysis of mRNA expression in peripheral CD14^+^ monocytes in healthy volunteers, standard care patients with infection, and intensive care patients with and without sepsis. (**A**) Principal component analysis (PCA) after variance stabilizing transformation of the count data. (**B**) Clustered heatmap of the standardized mRNA expression of genes measured by NanoString analysis. Sample clustering was separately performed for each patient cohort. Sample sizes: healthy controls (HC) *n* = 54, standard care (SC) *n* = 42, intensive care unit (ICU) *n* = 76, no sepsis *n* = 40, and sepsis *n* = 36.

From the RNA sequencing analysis, 55 highly variable genes (previously described to delineate activation profiles of monocytes [38]) were selected for an investigation of the complete patient cohorts (see Figure 3A). In brief, a custom-designed panel was generated that allowed a quantitative gene expression analysis of isolated circulating CD14^+^ blood monocytes in a mini-array by using the NanoString technology [39]. In total, isolated circulating monocytes from *n* = 76 ICU patients (*n* = 36 with sepsis, *n* = 40 without sepsis), *n* = 42 standard care patients, and *n* = 54 healthy controls were analyzed regarding their gene expression profile. Principal component analysis (PCA) revealed dense clustering of the healthy control samples (Figure 5A). Samples from SC (with infections) and from ICU sepsis patients were more dispersed, with septic patients displaying the most variance. Visualizing the expression of mRNAs in a clustered heatmap supported the very homogeneous population of healthy individuals compared to the heterogenic SC and ICU cohorts (Figure 5B). MRNAs in the largest cluster (cluster I) showed a very high expression in septic patients compared to the other three cohorts. This cluster contains mRNAs encoding for cytokine and chemokine receptors (ACKR3/CXCR7, IL1R2, and IL18R1), and metalloproteinases (MMP8, MMP9, and ADAMTS2), but also proteins with anti-microbial activity (ADM, LTF, LCN2, MPO, and DEFA1). Most of the mRNAs in cluster II encode for inflammatory chemokines or cytokines (e.g., CXCL2, IL-1β, and TNF). The expression of these mRNAs among the different patients and the healthy volunteers was very heterogeneous and not exclusive to one cohort. Proteins encoded by cluster III mRNAs are involved in adhesion (CD38, FCGR3B, and SLAMF7) and the classical complement system (C1QB and C1QC). Like in cluster I, mRNAs in cluster III displayed a high expression among septic patients with respect to the other three cohorts. Cluster IV contains mRNAs with a moderate expression in healthy volunteers, but reduced expression among the patient cohort, with septic patients showing the least expression. Proteins encoded by mRNAs from this cluster are very diverse in their functions. Longitudinal analysis showed a decline in the expression of most of the mRNAs analyzed (Supplementary Materials Figure S3). mRNAs from clusters I and IV showed the highest expression upon ICU admission and declined on day 3 and day 7 during the ICU stay. These clusters contain mRNAs encoding for anti-microbial effectors, metalloproteinases, chemokines, and cytokines, as well as their receptors. On the other hand, mRNAs in cluster III showed an increase in expression until day 7. This cluster is largely identical to cluster IV from Figure 5B, containing mRNAs with different functions. For septic patients, no substantial changes in mRNA expression patterns could be identified (Supplementary Materials Figure S3).

**Figure 6:**
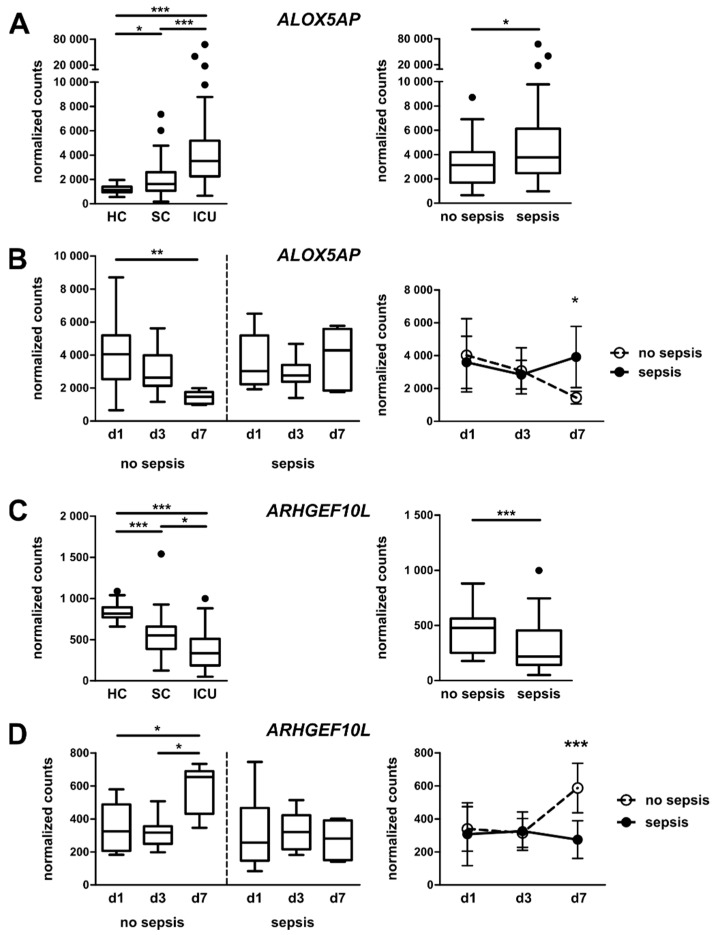
mRNA expression in peripheral CD14^+^ monocytes in healthy volunteers, standard care patients with infection, and intensive care patients with and without sepsis. (**A**) Expression of arachidonate 5-lipoxygenase-activating protein (ALOX5AP) mRNA in CD14^+^ monocytes from healthy volunteers, standard care patients with infection, and intensive care patients (left panel) or ICU patients with and without sepsis (right panel). (**B**) Longitudinal assessment of ALOX5AP mRNA expression in CD14^+^ monocytes from patients on day 1, 3, and 7 of the ICU stay. (**C**) Expression of monocytic guanine nucleotide exchange factor 10-like protein (ARHGEF10L) mRNA in CD14^+^ monocytes from healthy volunteers, standard care patients with infection, and intensive care patients (left panel) or ICU patients with and without sepsis (right panel). (**D**) Longitudinal assessment of ARHGEF10L mRNA expression in CD14^+^ monocytes from patients on day 1, 3, and 7 of the ICU stay. Statistics: * indicates *p* < 0.05, ** *p* < 0.01, and *** *p* < 0.001. For a comparison of two groups, the Mann–Whitney U test was used; for a comparison of more than two groups, the Kruskal–Wallis test was performed, followed by post hoc testing by Dunn’s multiple comparison test; and for longitudinal analysis (Figure 6B,D, right panels), two way ANOVA was performed, followed by a Bonferroni post-test. Sample sizes: healthy controls (HC) *n* = 54, standard care (SC) *n* = 42, intensive care unit (ICU) *n* = 76, no sepsis *n* = 40, and sepsis *n* = 36; no sepsis: d1 = 14, d3 = 10, and d7 = 6; sepsis: d1 = 16, d3 = 13, and d7 = 6.

Out of the 55 selected mRNAs from the NanoString analysis, 30 mRNAs showed significant differences in their expression between ICU patients with sepsis and ICU patients without sepsis (for example, see Figure 6A,C, right panels). From the 30 mRNAs, nine showed significant changes when comparing day 1 and day 7 of the ICU stay (for example, see Figure 6B,D, left panels). Only two mRNAs showed significant differences when comparing their expressions on day 7 of the ICU stay from ICU patients with and without sepsis (Figure 6B,D, right panels). After correcting for multiple comparisons by Bonferroni correction using an adjusted significance level of α = 0.05 divided by 55, ARHGEF10L remained significant, while ALOX5AP did not. In detail, the mRNA encoding arachidonate 5-lipoxygenase-activating protein (ALOX5AP, FLAP) was highly upregulated in monocytes from ICU patients compared to healthy controls and in septic patients compared to non-septic patients (Figure 6A). Moreover, longitudinal analyses on days 1, 3, and 7 of the ICU stay revealed a normalization of ALOX5AP mRNA expression from non-septic patients towards the expression level of healthy individuals, whereas the mRNA levels from patients with sepsis remained elevated (Figure 6B). ALOX5AP is important for leukotriene synthesis and is considered a marker for GC/TGF-β-activated macrophages [40]. In contrast, the mRNA encoding for the Rho guanine nucleotide exchange factor (GEF) 10-like protein (ARHGEF10L) decreased in ICU patients compared with healthy controls and was even less abundant in patients with sepsis compared to non-septic patients (Figure 6C). After seven days of ICU stay, the mRNA expression from non-septic patients rose again towards conditions in healthy controls, but mRNA levels from septic patients remained low (Figure 6D). ARHGEF10L, also known as GrinchGEF, is a Rho-specific guanine nucleotide exchange factor [41]. It has to be kept in mind that only patients that did not die within the first days (‘worst prognosis’) and were not transferred from the ICU to the SC ward (‘best prognosis’) provided samples for a longitudinal assessment of monocyte activation patterns. However, patients with full longitudinal sampling did not differ from patients without follow-up measurements regarding disease severity (i.e., APACHE II score) or outcome (i.e., ICU or in-hospital mortality) (Supplementary Materials Table S1).

### 3.5. Monocytic ALOX5AP and ARHGEF10L Expression as Markers of Prognosis for ICU Patients

**Figure 7:**
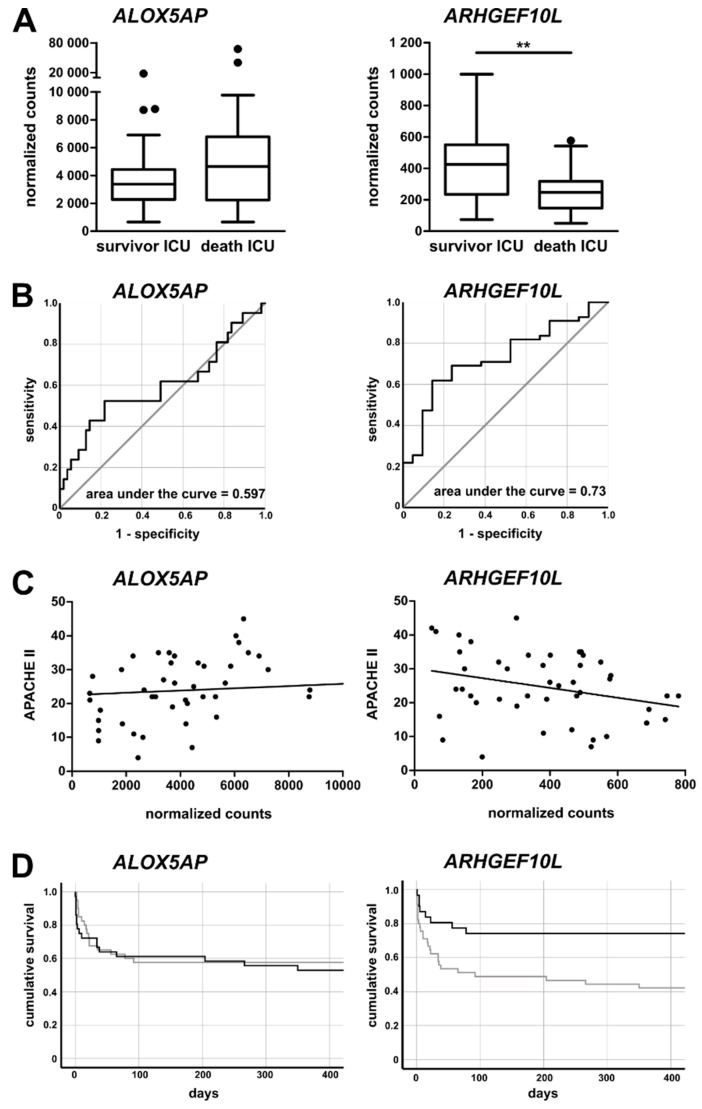
Correlation of mRNA expression with survival and prognosis for intensive care patients. (**A**) Expression of ALOX5AP and ARHGEF10L mRNA in CD14+ monocytes from intensive care patients surviving and not surviving their ICU stay. (**B**) Receiver operating characteristic (ROC) curve analysis. (**C**) Correlation analysis of APACHE II score with mRNA expression of ALOX5AP in monocytes from ICU patients (left panel, three values out of range are included in the analysis, but not displayed in the graph) and ARHGEF10L (right panel). (**D**) Kaplan–Meier survival curves for ICU patients. Left: ALOX5AP expression <3681.75 counts (grey line) or ≥3681.75 counts (black line), Log rank 0.238, *p* = 0.625; Right: ARHGEF10L expression < 413,265 counts (grey line), black ≥ 413,265 counts (black line), Log rank 6.978, *p* = 0.008. Statistics: ** indicates *p* < 0.01. For a comparison of two groups, the Mann–Whitney U test was used. Sample sizes: survivor ICU *n* = 55 and death ICU *n* = 21.

To further validate the clinical relevance of the two mRNAs for ICU patient prognosis, we analyzed their respective expression by comparing patients surviving the ICU stay with patients that died at the ICU. For ALOX5AP, no significant differences were observed, but there was a trend towards increased mRNA expression in patients who died during the ICU stay (Figure 7A, left panel). This was corroborated by receiver operating characteristic (ROC) curve analysis for ALOX5AP mRNA expression, which did not reveal a high prognostic value (Figure 7B, left panel). On the other hand, the expression of ARHGEF10L mRNA was significantly decreased among patients who died in the ICU, and the ROC curve analysis revealed a good performance as a discriminative prognostic marker (Figure 7A,B, right panels). Furthermore, we found a clear correlation between the APACHE II score for ICU patients and ALOX5AP mRNA expression (r = 0.4253, *p* = 0.0032; Figure 7C), supporting its association with disease severity. ARHGEF10L mRNA expression, on the other hand, tended to decrease with higher APACHE II scores (r = -0.2819, *p* = 0.0577; Figure 7C). We calculated Cox regressions with respect to survival for both mRNAs (omnibus test of model coefficients, ALOX5AP *p* = 0.004 and ARHGEF10L *p* = 0.007 for variables in the equation). Using the Youden index, we calculated optimal cutoff values for both mRNAs. Using Kaplan–Meier curve analysis, the chances for the survival of ICU patients was displayed. For ALOXAP5, no improved outcome was detected, as survival curves were almost identical. However, patients with ARHGEF10L expression equal to or above the cutoff showed an almost doubled chance of survival compared to patients with expression below the threshold (Figure 7D).

## 4. Discussion

In this study, we comprehensively analyzed the mRNA expression profiles in CD14^+^ monocytes from critically ill patients and evaluated the usability of regulated monocytic mRNAs as prognostic markers, particularly in sepsis. In addition, multicolor flow cytometry revealed alterations in the composition of circulating monocyte subsets and their downregulation of HLA-DR and CX_3_CR1 surface expression in sepsis. Comparing mRNA expression by full RNA sequencing in CD14^+^ monocytes from septic and non-septic patients showed differences in the immune response, metabolism, and processes associated with the cell cycle. An in-depth analysis of the patient cohorts revealed that in sepsis, the mRNA encoding ALOX5AP is upregulated and persists over a period of at least seven days of the ICU stay. ARHGEF10L mRNA displays an opposing expression pattern with prolonged downregulation in septic patients. Furthermore, ARHGEF10L mRNA expression negatively correlates with disease severity and patient survival.

Circulating monocytes are key players of the innate immune system and are involved in an acute response to, e.g., bacterial infections. In most cases, three different subpopulations of blood monocytes are distinguished: CD14^+^CD16^−^ classical monocytes, CD14^−^CD16^+^ non-classical monocytes, and an CD14^+^CD16^+^ intermediate subtype [22]. The non-classical and intermediate monocytes display more pro-inflammatory properties, while the classical monocytes are more immature and phagocytic [22]. However, intermediate monocytes are reported to be a more heterogeneous population containing cells with cytotoxic features [21]. In our study, we discriminated between these three extensively described subpopulations, but also included another “intermediate” population (CD14^low^CD16^−^, as displayed in Figure 1B).

A decrease in the abundance of classical monocytes for septic patients has been reported, alongside an increase in the proportions of intermediate and non-classical monocytes [22]. Similar results were obtained from a study analyzing low-grade inflammation after the induction of experimental endotoxemia in humans [42]. The results from our study are in line with prior observations; however, changes were rather moderate when comparing no sepsis and sepsis (see Figure 2B). A possible explanation for this could be the different gating strategy employed by Mukherjee and colleagues, including pre-selection on HLA-DR^+^ cells [22]. In fact, we observed a tendency towards a decreased surface expression of HLA-DR and CX_3_CR1 in our four subpopulations, reflecting previous findings [19,23,24,25]. Consistently, reduced HLA-DR expression on circulating monocytes was also observed by others, who could additionally demonstrate that the persistent suppression of HLA-DR expression predicts the outcome of patients [3,4].

We sought to analyze the whole transcriptome of the major monocyte subpopulation and therefore isolated CD14^+^ monocytes (corresponding to the classical and intermediate subpopulations) from the whole blood of randomly selected patients with sepsis, without sepsis, and healthy individuals. RNA sequencing revealed a clear separation of samples from healthy individuals and samples from ICU patients (see Figure 3B). A recent study nicely demonstrated widespread changes in the methylome of circulating monocytes from septic patients with the acquisition of a tolerized phenotype and organ dysfunction [43].

We compared data from our isolated monocytes to distinct activation programs of stimulated human monocyte-derived in vitro-differentiated macrophages, as comprehensively presented in [38]. The enrichment of transcriptional activation signatures differed between monocytes from healthy controls and ICU patients with or without sepsis (Figure 4A). Interestingly, activation signatures from monocytes of ICU patients showed enrichment in the GC-related module, which could reflect their reaction towards increased endogenous cortisol as part of the stress response. Sepsis patients showed an overall increased enrichment in pro-inflammatory signatures compared to non-septic healthy controls. Increased enrichment was observed in activation profiles related to stimulation with combinations of P3C/PGE_2_/TNF-α. Notably, TPP (TNF/P3C/PGE_2_) signaling has been linked to chronic inflammation [38].

A more in-depth analysis of significantly differentially expressed genes comparing non-sepsis with septic patients revealed an enrichment of pathways associated with metabolism and glucose homeostasis, the immune response, and the negative regulation of proliferation and differentiation (see Figure 4C). During sepsis, monocytes undergo a phenotypical transition from the hyperinflammatory to the immunotolerant state, which is accompanied by shifts in cellular metabolism and cytokine production [15]. Blocking of the cell cycle in CD14^+^ monocytes is in line with the reduction of classical monocytes in sepsis observed by FACS analysis (see Figure 2B). For neutropenia during sepsis, the reduction of neutrophils could be attributed to apoptosis, as well as a sustained blockade of hematopoietic stem cell (HSC) differentiation [44].

Similar to the PCA derived from the RNA sequencing data (as shown in Figure 3B), PCA from NanoString data showed a homogeneous cohort of healthy individuals, while ICU patients were more scattered (with sepsis patients being the most widespread); the SC patients displayed in-between characteristics (see Figure 5A). The clustering of genes in a heatmap showed distinct groups of genes with similar expression patterns in the cohorts. Proteins encoded by mRNAs in cluster I—with the highest expression in septic patients—function in inflammatory processes, like anti-microbial responses and the recognition of chemokines and cytokines. However, some mRNAs also point to an anti-inflammatory polarization of monocytes: metalloproteinases, as well as MRC1, the mannose receptor. In addition, TNFAIP6 mRNA has been demonstrated to function in macrophage transition from a pro- to anti-inflammatory phenotype [45], possibly linking the dichotomous pattern observed in cluster I. Similarly, cluster III mRNAs contribute to defense responses (complement and adhesion), and the chemokine receptor CCR2 is typically expressed on inflammatory monocytes. Regarding the longitudinal analysis of non-septic patients, most mRNAs related to inflammatory processes were downregulated from admission to day 7 of their ICU stay. This may reflect a recovery of patients without diagnosed sepsis. For septic patients, mRNA expression was heterogeneous, but generally higher than in patients without sepsis (Supplementary Materials Figure S3). Therefore, those monocytes do not significantly change their phenotype over the time period observed, which is also evident from the analysis of single mRNAs (Figure 6B,D). This phenomenon may be attributed to the generally more overstrained immune system and the inability to fully regenerate. Patients with sepsis demonstrated very consistent and persistent changes (at least within the observed period), as also described in [3,4], but this may be confounded by a greater severity of illness that may also affect the immune cell phenotype.

In order to screen the selected 55 candidates for potential prognostic markers, we identified two mRNAs with interesting expression patterns. First, the mRNA encoding for ALOX5AP seems to more present in critically ill patients. Furthermore, even after seven days in the ICU, patients with sepsis failed to restore ALOX5AP mRNA expression to baseline conditions, unlike patients with excluded sepsis (Figure 6A,B). Leukotrienes are potent inflammatory mediators involved in the inflammatory response in, e.g., asthma and sepsis. The synthesis of leukotrienes from arachidonic acid is initiated by 5-lipoxygenase (5-LO), together with ALOX5AP. Despite lacking enzymatic activity, ALOX5AP is able to bind to arachidonic acid, thereby transferring it to 5-LO [46]. In monocytes, inflammatory stimuli like LPS or TNF-α induce ALOX5AP expression [47,48]. This implies that leukotrienes are associated with the severity of disease, which was further corroborated by the findings that ICU survivors express less ALOX5AP mRNA (Figure 7). The second identified mRNA, encoding ARHGEF10L, was found to be downregulated in sepsis and expression remained low during the ICU stay of septic patients (Figure 6C,D). Furthermore, patients not surviving ICU expressed significantly lower amounts of that mRNA and the ROC analysis revealed a moderate diagnostic ability (AUC = 0.73) of that marker (Figure 7B). Importantly, the chance of survival was improved when patients expressed higher amounts of monocytic ARHGEF10L mRNA. Besides a described role in hepatocellular tumorigenesis [49], not much is known about the function of ARHGEF10L. It has been demonstrated that ARHGEF10L specifically interacts with RhoA, RhoB, and RhoC, but not with other members of the Rho family of small GTPases [41]. The stimulation of RhoA leads to a reorganization of the actin cytoskeleton of the cell, and may be implicated in cell division.

## 5. Conclusions

Our comprehensive analysis of circulating monocytes in critically ill patients revealed a distinct activation pattern, particularly in ICU patients with sepsis, which resembles characteristic functional stages of monocyte-derived macrophages. The association with disease severity, the longitudinal recovery or lack thereof during the ICU stay, and the association with prognosis indicate the clinical relevance of the monocytic gene expression profile during sepsis. In particular, the persistently reduced monocytic mRNA expression of ARHGEF10L was linked to reduced survival. Its value as a prognostic marker or therapeutic target should be addressed in further studies.

## Figures and Tables

**Table 1:** Characteristics of the different study cohorts comprising healthy volunteers (HC); standard care patients with bacterial infections (SC); and intensive care unit patients (ICU), with or without sepsis.

Parameter	HC	SC	ICU	ICU: No Sepsis	ICU: Sepsis
Number, *n*	54	42	76	40	36
Male/female, *n*	30/24	31/11	45/31	24/16	21/15
Age (years)	48.5 (24–77)	65.5 (21–88)	68 (18–97)	60.5 (23–92)	71 (18–97)
Days in hospital	-	6.5 (3–25)	14 (1– 97)	13 (2–89)	20 (1–97)
Days on ICU	-	-	4 (1–79)	4 (1–37)	4 (1–79)
Death on ICU, *n* (%)	-	-	21 (27.6%)	5 (12.5%)	16 (44.4%)
Death in hospital, *n* (%)	-	2 (4.8%)	28 (36.8%)	10 (25%)	18 (50%)
APACHE II score	-	-	22.5 (2–45)	20 (2–43)	25.5 (9–45)
Leukocytes (per nL)	5.8 (3.8–10.0)	9.4 (2.1–23.0)	13.5 (0.5–42.9)	10.7 (2.7–31.4)	15.5 (0.5–42.9)
Monocytes (per nL)	0.50 (0.25–0.95)	0.71 (0.06–1.86)	0.62 (0–3.45)	0.66 (0.01–3.45)	0.52 (0–2.16)
IFN-γ, (pg/mL)	4.21 (0–500)	8.07 (0–372)	10.6 (0–527)	9.23 (0–101)	20.5 (0–527)
IL-6 (pg/mL)	0.36 (0.2–200)	9.25 (0.32–526)	137 (2.4–500,000)	56.5 (8.88–1490)	204 (2.4–500,000)
IL-8 (pg/mL)	4.53 (1.71–33.8)	7.47 (1.86–81.7)	23.3 (0–1000)	14.8 (0–282)	29.3 (3.18–1000)
TNF-α (pg/mL)	0.57 (0–63.3)	0.63 (0–87.2)	1.51 (0–126)	1.42 (0–61.9)	1.56 (0–126)
Cholesterol (mg/dL)	-	-	123 (41–374)	123 (41–374)	128 (60–223)
Triglyceride (mg/dL)	-	-	139 (40–434)	148 (40–434)	128 (63–302)
Site of infection, *n* (%)					
Pulmonary	-	14 (33.3%)	17 (22.4%)	-	17 (47.2%)
Urinary	-	17 (40.5%)	5 (6.6%)	-	5 (13.9%)
Abdominal	-	6 (14.3%)	12 (15.8%)	-	12 (33.3%)
Bloodstream	-	2 (4.8%)	1 (1.3%)	-	1 (2.8%)
Other	-	3 (7.1%)	1 (1.3%)	-	1 (2.8%)
Culture positive, *n* (%)	-	11 (26.2%)	21 (27.6%)	-	21 (58.3%)
Gram neg., *n*	-	3	7	-	7
Gram pos., *n*	-	7	10	-	10
Gram pos. and neg., *n*	-	0	3	-	3
Fungal, *n*	-	1	1	-	1

The median and range are given, unless indicated otherwise.

## Supplementary Materials

The following are available online at https://www.mdpi.com/2077-0383/9/1/127/s1, Figure S1: Monocyte subsets in healthy volunteers, standard care patients with infection, and intensive care patients; Figure S2: Monocyte subsets in ICU patients with and without sepsis; Figure S3: NanoString analysis of mRNA expression in peripheral CD14^+^ monocytes in intensive care patients with and without sepsis on day1, day 3, and day 7 of the ICU stay; Table S1: Characteristics of intensive care unit patients (ICU) with or without follow-up measurements.
