# Supplementary material for: Differential Gene Expression in Circulating CD14+ Monocytes Indicates the Prognosis of Critically Ill Patients with Sepsis
Source: J Clin Med. 2020 Jan 2;9(1):127. doi: 10.3390/jcm9010127 (PMC7019484; doi:10.3390/jcm9010127)
Supplement: Supplementary file 1 [file jcm-09-00127-s001.pdf]

Supplementary Material

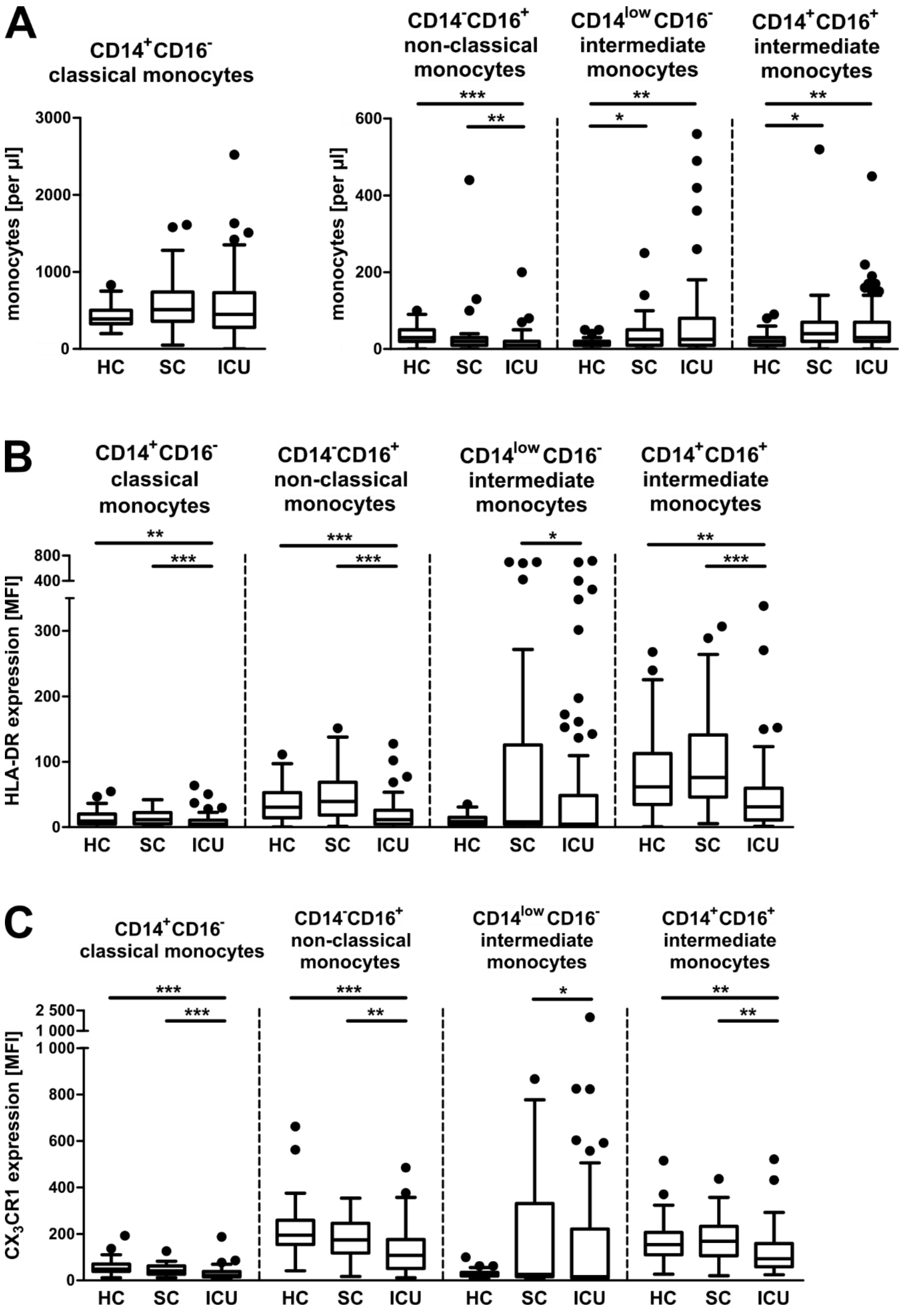

Supplementary Figure S1. Monocyte subsets in healthy volunteers, standard care patients with infection, and intensive care patients. (**A**) Absolute numbers of monocyte subpopulations from the different patient cohorts form HC, SC and ICU. (**B**) and (**C**) Mean fluorescence intensity (MFI) of HLA-DR (**B**) and CX_3CR1 (**C**) expression of the monocyte subpopulations in HC, SC and ICU patients. Statistics: * indicates $p < 0.05$, ** $p < 0.01$, *** $p < 0.001$. For comparison of more than two groups the Kruskal–Wallis test was performed followed by a post hoc test by Dunn´s multiple comparison test. Sample sizes: healthy controls (HC) $n = 54$, standard care (SC) $n = 42$, intensive care unit (ICU) $n \geq 75$.

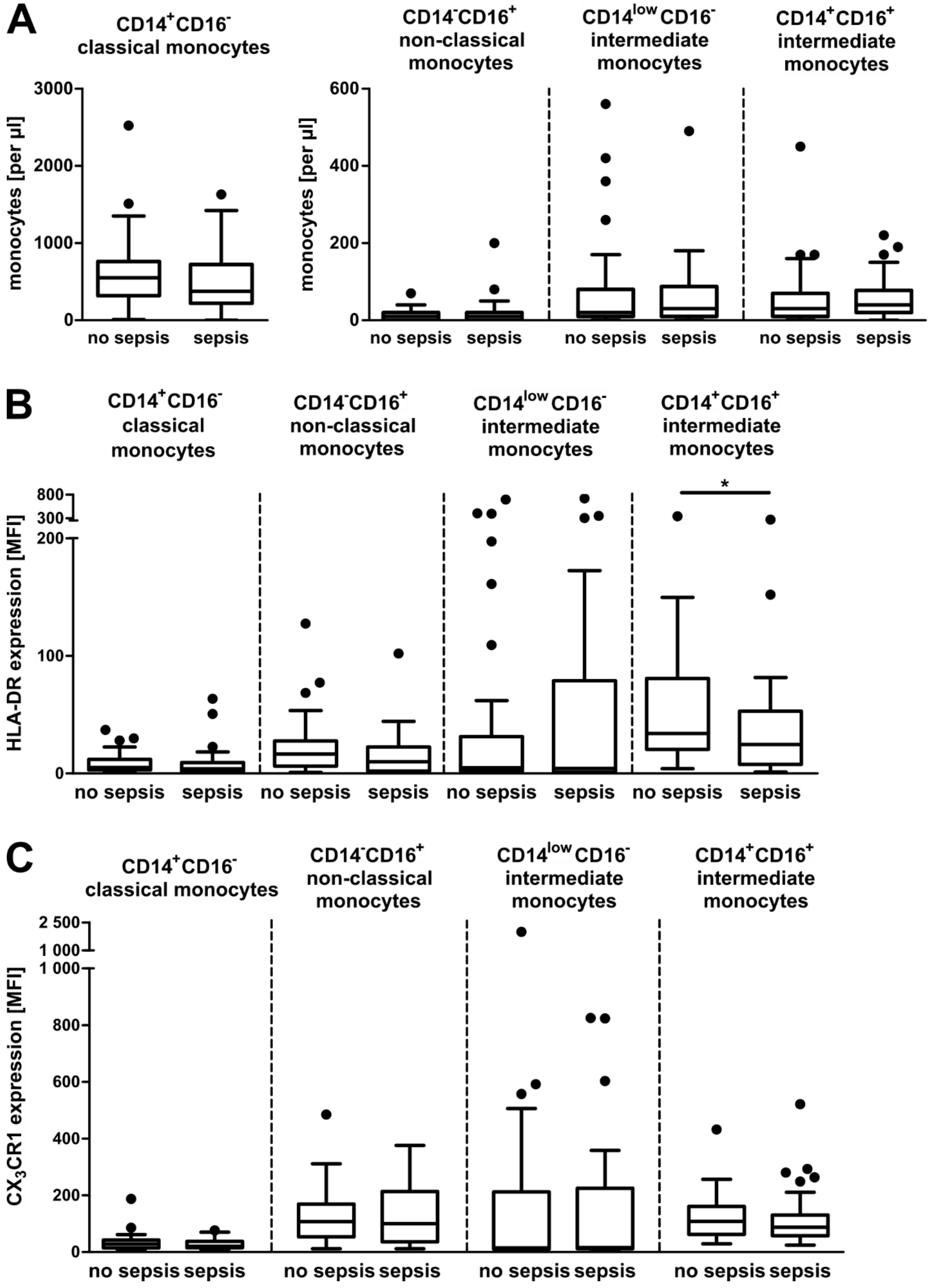

Supplementary Figure S2. Monocyte subsets in ICU patients with and without sepsis. (**A**) Absolute numbers of monocyte subpopulations from ICU patients with and without sepsis. (**B**) and (**C**) Mean fluorescence intensity (MFI) of HLA-DR (**B**) and CX_3CR1 (**C**) expression of the monocyte subpopulations in ICU patients with and without sepsis (**C**). Statistics: * indicates $p < 0.05$. For comparison of two groups, the Mann–Whitney U test was used. Sample sizes: no sepsis $n \geq 39$, sepsis $n = 36$.

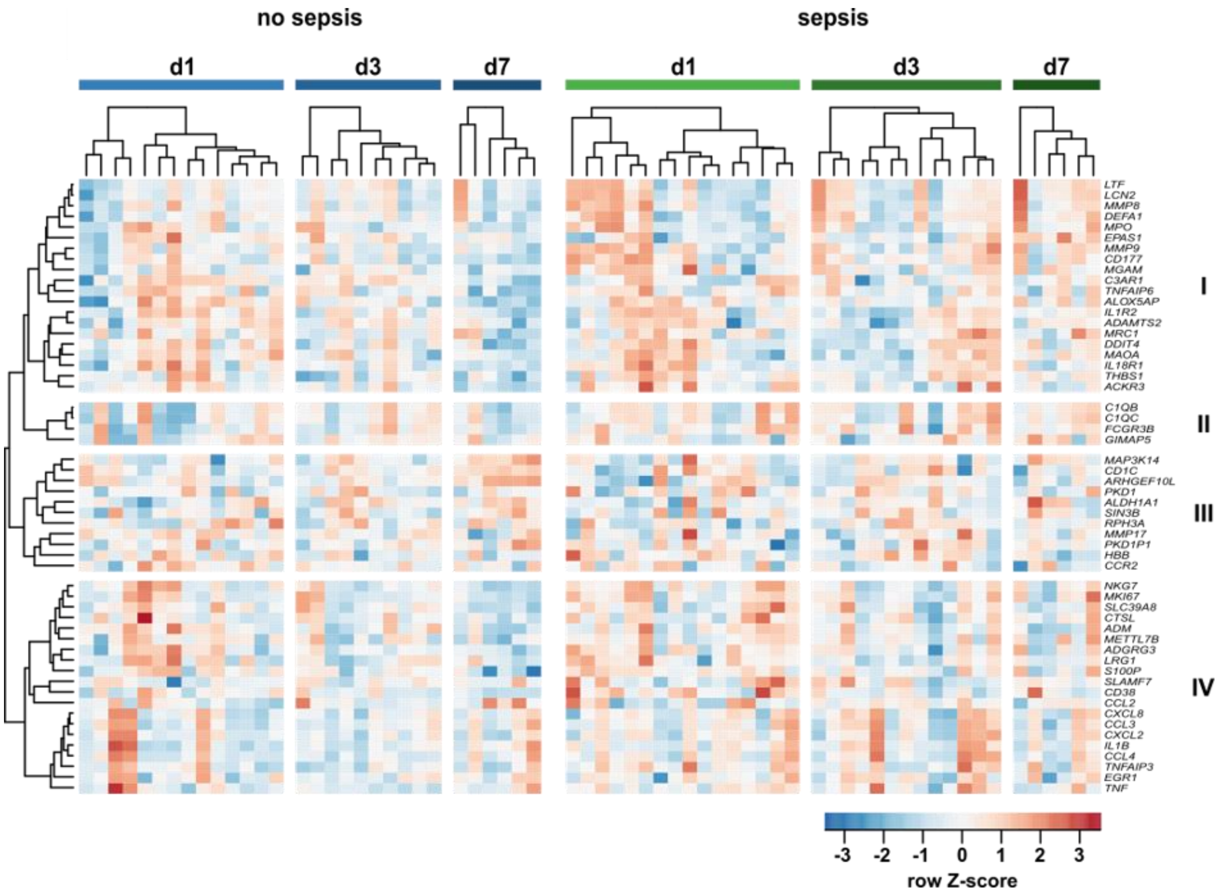

Supplementary Figure S3. NanoString analysis of mRNA expression in peripheral $CD14^+$ monocytes in intensive care patients with and without sepsis on day1, day 3 and day 7 of ICU stay. Clustered Heatmap of the standardized mRNA expression of genes measured by NanoString analysis. Sample clustering was separately performed for each group. Sample sizes: no sepsis: d1 = 14, d3 = 10, d7 = 6; sepsis: d1 = 16, d3 = 13, d7 = 6.

Supplementary Table S1. Characteristics of intensive care unit patients (ICU) with or without follow-up measurements.

Parameter	No Follow-Up Measurement	Follow-Up Measurement	*p*-Value
Number, *n*	46	30	-
Male/female, *n*	28/18	17/13	n.s.
Age (years)	71.5	64	n.s.
Days in hospital	11	22	0.001 **
Days on ICU	3	12	<0.001 ***
Death on ICU, *n* (%)	13 (28.3%)	8 (26.7%)	n.s.
Death in hospital, *n* (%)	16 (34.8%)	12 (40%)	n.s.
APACHE II score	22	23	n.s.
Leukocytes (per nL)	10.7	16.1	0.012 *
Monocytes (per nL)	0.62	0.62	n.s.

The median is given, unless indicated otherwise. Statistics: * indicates $p < 0.05$, ** $p < 0.01$, *** $p < 0.001$. For comparison between two groups, Mann–Whitney U test (for continuous variables) or Pearson Chi-squared test (for nominal variables) was used. N.s.: not significant.
